# Osteoporosis management in Australian general practice: an analysis of current osteoporosis treatment patterns and gaps in practice

**DOI:** 10.1186/s12875-020-01103-2

**Published:** 2020-02-12

**Authors:** Pradnya Naik-Panvelkar, Sarah Norman, Zain Elgebaly, Jeff Elliott, Allan Pollack, Jill Thistlethwaite, Clare Weston, Markus J. Seibel

**Affiliations:** 1 NPS MedicineWise, Level 7, 418A, Elizabeth Street, Surry Hills, NSW 2010 Australia; 2 Department of Endocrinology & Metabolism, Concord Repatriation General Hospital, The University of Sydney and Bone Research Program, ANZAC Research Institute, Concord, NSW 2139 Australia

**Keywords:** Osteoporosis, Primary care, Treatment patterns, Practice gaps, GP perspectives, Bisphosphonates, Denosumab, Adherence

## Abstract

**Background:**

Among Australians aged 50 and over, an estimated 1 in 4 men and 2 in 5 women will experience a minimal trauma fracture during their remaining lifetime. Effective fracture prevention is hindered by substantial undertreatment, even of patients who clearly warrant pharmacological therapy. Poor adherence to osteoporosis treatment is also a leading cause of repeat fractures and hospitalisation. The aim of this study was to identify current osteoporosis treatment patterns and gaps in practice in Australia, using general practice data, and to explore general practitioners’ (GPs’) attitudes to osteoporosis treatment and their views on patient factors affecting osteoporosis management.

**Methods:**

The study was conducted in two phases. Phase 1 was a longitudinal retrospective cohort study which utilised data from MedicineInsight – a national general practice data program that extracts longitudinal, de-identified patient data from clinical information systems (CISs) of participating general practices. Phase 2 included semi-structured, in-depth telephone interviews with a sample of MedicineInsight practice GPs. Data were analysed using an inductive thematic analysis method informed by the theory of planned behaviour.

**Results:**

A diagnosis of osteoporosis was recorded in 12.4% of patients over the age of 50 years seen in general practice. Of those diagnosed with osteoporosis, almost a quarter were not prescribed osteoporosis medicines. From 2012 to 17, there was a progressive increase in the number of denosumab prescriptions, while prescriptions for bisphosphonates and other osteoporosis medicines decreased. More than 80% of patients who ceased denosumab treatment had no subsequent bisphosphonate prescription recorded. Interviews with GPs revealed beliefs and attitudes that may have influenced their intentions towards prescribing and osteoporosis management.

**Conclusions:**

This study suggests that within the Australian general practice setting, osteoporosis is underdiagnosed and undertreated. In addition, it appears that most patients who ceased denosumab treatment had no record of subsequent antiresorptive therapy, which would place them at risk of further fractures. The study supports the need for the development of clinical education programs addressing GP knowledge gaps and attitudes, and the implementation of specific interventions such as good reminder/recall systems to avoid delays in reviewing and treating patients with osteoporosis.

## Background

Osteoporosis – a chronic condition characterised by skeletal deterioration and an increased risk of fractures – is a common and costly public health problem [1]. Minimal trauma fractures (MTFs) are associated with significant morbidity and mortality [2]. The prevalence of osteoporosis increases with age in both men and women [3]. The 2012 Burden of Disease Report [4] estimated that 4.74 million Australians over the age of 50 had osteoporosis or poor bone health, and this figure was predicted to increase to 6.2 million by 2022. It is estimated that among Australians aged 50 and over, 1 in 4 men and 2 in 5 women will experience a minimal trauma fracture during their remaining lifetime [3].

Timely diagnosis and appropriate pharmacological management of osteoporosis have been shown to reduce fracture rates [5]. According to the Royal Australian College of General Practitioners (RACGP) osteoporosis guidelines, bisphosphonates and denosumab are first-line medicines for the prevention of fractures and repeat fractures in patients with osteoporosis [6]. In Australia, for patients to receive subsidised medicines to treat osteoporosis, the condition must have been diagnosed following a MTF or based on a bone mineral density (BMD) T-score of − 2.5 or less for patients aged 70 years or older [7].

In Australia, general practitioners (GPs) are the most likely first point of contact for health-related concerns, including the diagnosis and treatment of osteoporosis and are the main prescribers of drug therapy for osteoporosis [8]. Individuals hospitalised due to fragility fractures are referred to their GPs by hospitals to ensure continuity of care. Fracture liaison services do operate in some parts of Australia and their findings and recommendations about osteoporosis treatment are also communicated to the patient’s GP [9]. However, it must be noted that in Australia patients do not register with a particular general practice and may receive treatment from any practice they choose to attend.

Despite remarkable advances in our understanding of the pathogenesis and treatment of osteoporosis, the disease is still underdiagnosed and undertreated, even in patients who clearly warrant pharmacological therapy (i.e. those with incident MTFs) [10, 11]. Failure to prevent repeat fracture is one of the largest gaps in the practice of evidence-based medicine in Australia [1, 12]. Fewer than 20% of patients presenting with MTFs to hospitals or general practices are investigated or treated for osteoporosis [1, 6]. The 2014–15 Australian National Health Survey reported that nearly 35% of patients with a prior diagnosis of osteoporosis had not consulted a GP or a specialist in the previous 12 months for their condition [13].

Among those who are prescribed medicines, poor adherence to osteoporosis treatment is a leading cause of repeat fractures and hospitalisation, with patients either not taking their treatment as prescribed (poor adherence) or discontinuing therapy within six months (lack of persistence) [14]. Patient concerns about rare side-effects of osteoporosis medicines and the perception of lack of clear evidence in support of their long-term efficacy are commonly reported reasons for low rates of patient adherence to and persistence with osteoporosis treatment [11, 15]. A 2004 retrospective analysis of dispensing data showed that only 57% of Australians with osteoporosis persisted with bisphosphonate treatment after 12 months [16]. The level of adherence to denosumab since its addition in 2010 to the Pharmaceutical Benefits Scheme (PBS), which lists medicines subsidised by the Australian Government, remains unknown. Adherence to denosumab is required for continued reduction of fracture risk, especially in the light of evidence of multiple vertebral fractures occurring after stopping denosumab [6, 17, 18].

In view of these concerns, and the lack of information about current patterns of osteoporosis treatment using bisphosphonates and denosumab, current levels of treatment cessation in practice and potential barriers to osteoporosis treatment in Australia, the aims of this study were:

1. to identify current trends in osteoporosis treatment patterns within Australian general practice, and
2. to explore GPs’ beliefs about and attitudes to osteoporosis treatment and their views and approaches to managing patient factors affecting osteoporosis management in Australia.

## Methods

The study was conducted in two phases:

- PHASE 1: A longitudinal retrospective cohort study which utilised data from MedicineInsight to achieve the first aim of the study - to estimate the prevalence of a recorded diagnosis of osteoporosis in general practice, and identify treatment patterns for osteoporosis medicines, including cessation and switching of treatment modalities.
- PHASE 2: In-depth interviews with a sample of GPs who work in MedicineInsight practices to achieve the second aim of the study.

## Phase 1

### Study design

This was a longitudinal retrospective cohort study using MedicineInsight data. The study period spanned 1 January 2011 to 31 May 2018. MedicineInsight is an Australian large-scale national general practice data program developed and managed by NPS MedicineWise, a not-for-profit organisation which provides evidence-based education and information to health professionals and consumers, with funding support from the Australian Government Department of Health [19, 20]. MedicineInsight’s data collection extracts longitudinal, de-identified patient data from participating practices’ clinical information systems (CISs), such as Best Practice and Medical Director 3. The MedicineInsight program collects data on patient demographics, practice encounters (not including progress notes), diagnoses, prescriptions, pathology tests and referrals. The database comprises records for an open cohort of patients (between 15 and 20% of the Australian population) from more than 3300 GPs in 705 recruited general practices across Australia.

The RACGP National Research and Evaluation Ethics Committee granted ethics approval for the MedicineInsight program (NREEC 17–017) in December 2017 for standard operations and uses of the MedicineInsight database including the ongoing collection of de-identified data.

### Study population

**Fig. 1:**
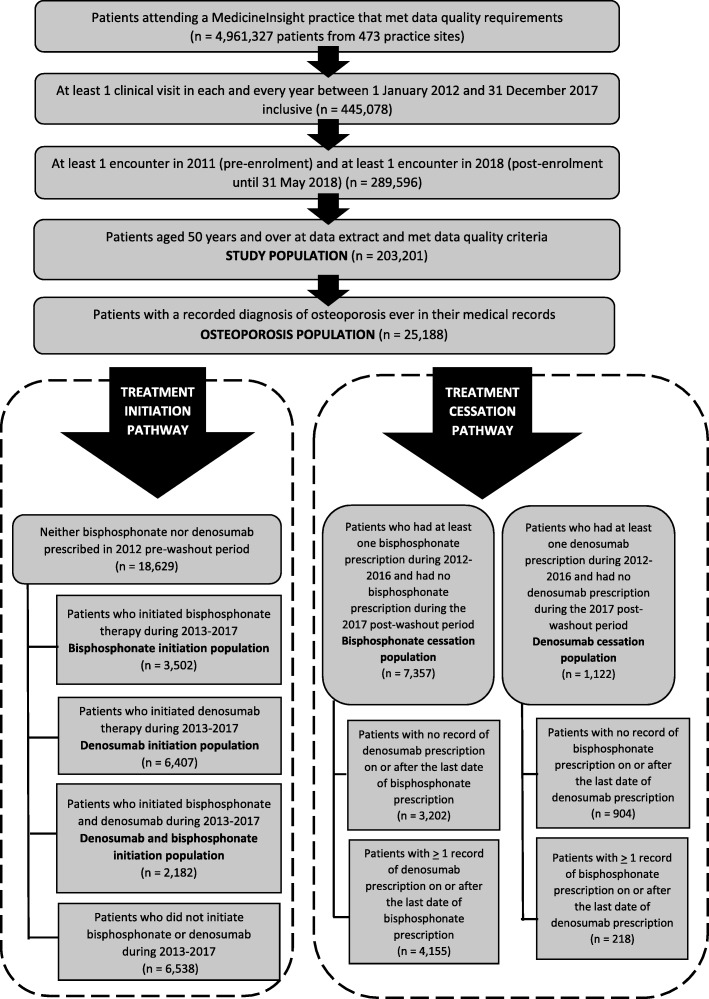
Study population selection process

Patients were selected for this study using the process described in Fig. 1.

Only patients who had a record of attending a MedicineInsight practice which met data quality requirements and who also fulfilled data quality criteria were selected for inclusion in the study population. MedicineInsight data quality requirements included that practices had been established for at least 2 years before the end of the analysis period and had no interruptions of longer than 2 months in practice data in the 2 years to the end of the analysis period. Patient data quality criteria included complete information for defined age, sex, and postcode, and at least one entry in one of the diagnosis fields (diagnosis, reason for encounter, and reason for prescription). Additionally, to reliably describe treatment patterns and cessation, patients had to be regularly attending the practice throughout the study period; this was defined as having had at least one clinical encounter per year every year during the treatment period and at least one encounter in the pre- and post- enrolment periods.

**Fig. 2:**
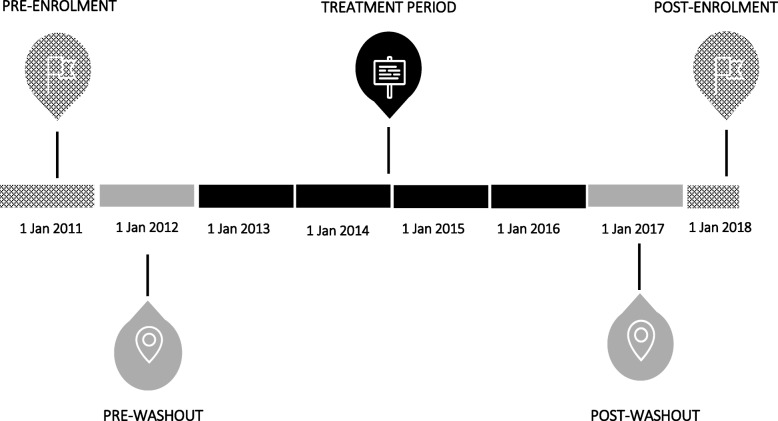
Study timeline, MedicineInsight 2011–2018

In order to infer ‘treatment initiation’ and ‘treatment cessation’ in our study population, we applied a 1-year pre-washout period (1 January 2012 to 31 December 2012) and 1-year post-washout period (1 January 2017 to 31 December 2017), during which time patients must have had no record of prescription of any of the osteoporosis medicines investigated, for inclusion in the study (Fig. 2). This assumes that patients who had no record of a prescription for an osteoporosis medicine during the 1-year pre-washout period had not taken osteoporosis medicines prior to the study period.

The osteoporosis population was selected from the study population as described in Fig. 1. We defined patients with a recorded diagnosis of osteoporosis as those who had codes (Docle or Pyefinch medical condition coding) or free text in the fields ‘diagnosis’, ‘reason for encounter’ or ‘reason for prescription’ that indicated osteoporosis at any time in their medical records. We did not use BMD results, which are largely unavailable through the MedicineInsight dataset, even if recorded in the patient notes. We did not use a record of prescription for osteoporosis medicines as part of our definition of a diagnosis of osteoporosis as some of these medicines can be also prescribed for other conditions, and because the use of osteoporosis medicines was one of the outcome variables of the study. Osteoporosis was defined by including many specific search terms and expressions (including spelling variations). Patients who met the above osteoporosis population criteria were further analysed for osteoporosis treatment patterns (Fig. 1).

Patients were defined as having had a prescription for osteoporosis medicines if they had at least one record of a prescription for denosumab, and/or medicines containing a bisphosphonate and/or ‘other’ osteoporosis medicines, including strontium ranelate, tibolone, raloxifene or teriparatide (either singly or in combination), during the treatment period.

Data collection was also limited to medicines for which a GP used the CIS to print a prescription for a patient. Only original (i.e. not repeat) prescriptions for osteoporosis medicines were examined, as this improved comparison of the number of original denosumab prescriptions (one every 6 months) with the number of original bisphosphonate prescriptions (one original and 5 repeats, covering a 6-month period) during the treatment period. ‘Original prescriptions’ in this context refers only to each individual prescription item, counted once, whether or not repeat prescriptions were simultaneously added to this item. ‘Original prescription’ does not refer only to the first or initial prescription that a patient may have received – it is a term used to distinguish these prescriptions from repeat prescriptions, which allow the patient to collect their medicine from the pharmacy without consulting a GP each time.

### Statistical methods

Analyses of the data were conducted using SAS version 9.4 (SAS Institute Inc., Cary, NC, USA), including the use of the SURVEYFREQ procedure. Descriptive statistics, frequencies, percentages (including characteristic-specific prevalence) and prevalence ratios were used to describe the cohort of patients and to present the study outcomes. To indicate the reliability of the estimates of prevalence and proportion, 95% confidence intervals (CIs) were calculated (a range of values that should contain the true measure 95% of the time), as were *p*-values. The 95% CIs were adjusted for clustering by general practice site.

## Phase 2

### Study design and population

Semi-structured telephone interviews, 45–60 min in duration, were conducted with a sample of GPs working within MedicineInsight practices. The interview guide was informed by a literature review and the results of Phase 1 and was developed in consultation with a GP employed by NPS MedicineWise and an external endocrinologist. The interview guide included open-ended questions focussing on exploring GP views about choice of osteoporosis medicines, use of different treatment patterns, and patient adherence to and cessation of osteoporosis treatment (See Additional file 1 for interview questions for GPs). The interview guide was piloted with an external GP to further improve validity and highlight any ambiguities.

An invitation to participate in the study was sent via electronic direct mail to GPs working in MedicineInsight practices who had previously consented to being contacted by NPS MedicineWise to participate in research studies. The invitation included links to the participant information sheet and consent form, which GPs were required to sign before participating in an interview. Interviews were conducted by one investigator at a time convenient to GPs. An incentive in the form of a voucher was provided to participating GPs for their time.

The qualitative GP study received ethics approval from Bellberry Human Research Ethics Committee (HREC 2018–07-533). Written informed consent for publication was obtained from participating GPs prior to conducting the interviews.

### Data analysis

Interviews were audio-recorded and content was transcribed verbatim. The data were aggregated to prevent individual GPs or their patients from being identified. The data were analysed using an inductive thematic analysis method [21], which allowed the development of themes from the original data.

We used the theory of planned behaviour (TPB) to help inform the analysis [22]. In the TPB, three important constructs influence the intention of a person (in this study the GPs) to adopt a given behaviour (osteoporosis management): attitude, subjective norm, and perceived behavioural control [22, 23]. Each of these constructs is influenced by a set of beliefs [24]. Intentions are thus affected by an individual’s perception of the potential positive or negative outcomes of the behaviour and, external to self, whether society (in this case professional peers and patients) is likely to approve (or disapprove) of that action and by an individual’s sense of how much control they have in a given situation to enact their desired behaviour. In clinical practice, external control is exerted by patients, families, and regulation bodies to name but a few.

Two investigators, JE and PN (pharmacists), independently coded half of the interview transcripts. From this, segments of text related to the research question were identified and labelled as initial ‘codes’, which were then grouped under higher order categories or ‘themes’. Codes and themes were then compared and refined until consensus was reached between the two investigators. The refined codes were then used for the remainder of the transcripts by one investigator, and the codes and themes were reviewed by the second investigator to ensure precision of data analysis. Any similarities and differences were discussed and amended by the two investigators until consensus was reached and a final thematic framework was generated. Data that did not fit under existing themes were coded as new codes and included as additional themes or subthemes after discussion with the study team. In line with the study aims above, only themes specifically related to GP barriers and gaps in osteoporosis treatment in Australia are discussed below.

## Results

### Phase 1

A total of 203,201 patients met the study inclusion criteria (Table 1). A diagnosis of osteoporosis was recorded for 25,188 of these patients, giving an overall prevalence of 12.4% (95% CI 11.7 to 13.0%). Significantly more women (17.6%) than men (5.3%) had a recorded diagnosis of osteoporosis, and this proportion increased with age in both genders (Table 1). The prevalence of a recorded diagnosis of osteoporosis was broadly consistent across quintiles of Socio-Economic Indexes for Areas (SEIFA). Regarding rurality, the proportion of patients with a recorded diagnosis of osteoporosis was lowest in remote areas (6.8%) and highest in major cities (13.0%).

**Table 1:** Patient sociodemographic characteristics and prevalence of a recorded diagnosis of osteoporosis, MedicineInsight 2012–17

CHARACTERISTICS		Total patient study population *N* = 203,201	Patients with osteoporosis recorded *N* = 25,188	Prevalence of recorded diagnosis of osteoporosis	
		Number	Number	Proportion (%)	95% CI
Sex	Male	86,221	4589	5.3	4.9–5.7
	Female	116,980	20,599	17.6	16.7–18.5
Age (years)	50–59	45,824	1090	2.4	2.2–2.6
	60–69	58,306	4033	6.9	6.5–7.3
	70–79	58,893	8730	14.8	14.0–15.6
	80–89	32,363	8544	26.4	25.1–27.7
	≥90	7815	2791	35.7	33.7–37.7
State/Territory	Australian Capital Territory	7003	755	10.8	8.7–12.8
	New South Wales	76,312	9954	13.0	12.1–14.0
	Northern Territory	247	10	4.0	0.9–7.2
	Queensland	31,961	4579	14.3	12.7–15.9
	South Australia	7962	947	11.9	9.6–14.2
	Tasmania	14,144	1335	9.4	8.0–10.8
	Victoria	34,903	4181	12.0	10.4–13.6
	Western Australia	30,668	3427	11.2	9.4–12.9
	*Missing*	1			
Rurality	Major city	128,498	16,755	13.0	12.3–13.8
	Inner regional	56,654	6722	11.9	10.5–13.2
	Outer regional	11,606	1153	9.9	8.3–11.6
	Remote/very remote	2690	183	6.8	5.4–8.2
	*Missing*	3753			
Socio-economic status (SEIFA^b^ IRSAD quintile)	1 (least advantaged)	32,553	4184	12.9	11.2–14.5
	2	32,372	3993	12.3	11.1–13.5
	3	53,017	6084	11.5	10.4–12.5
	4	35,848	4647	13.0	11.8–14.2
	5 (most advantaged)	48,839	6233	12.8	11.6–13.9
	*Missing*	572			

^b^Socio-Economic Indexes for Areas

### Trends in osteoporosis medicine prescribing

The study examined the number of original prescriptions in each subgroup of osteoporosis medicines – bisphosphonates, denosumab and other osteoporosis medicines – as a proportion of the total number of osteoporosis medicine prescriptions on record for the study cohort per calendar year, as well as the changes in the proportion of osteoporosis medicines prescribed compared to the total number of all medicines prescribed to the study cohort.

**Fig. 3:**
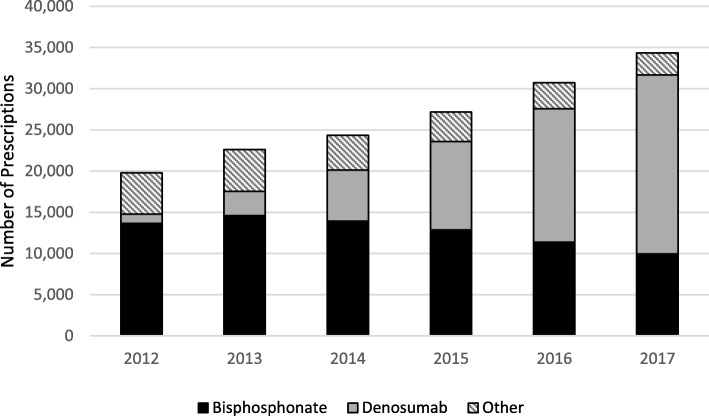
Osteoporosis medicine prescriptions per calendar year, 2012–2017

For osteoporosis medicines specifically, there was a steady increase in the total annual number of prescriptions for osteoporosis medicines, from just under 20,000 in 2012 to nearly 35,000 prescriptions in 2017 in this study cohort (Fig. 3). This was due to a progressive increase in the number of denosumab prescriptions, while at the same time prescriptions for both bisphosphonates and other osteoporosis medicines decreased (Fig. 3). Bisphosphonate and denosumab prescriptions together accounted for more than 85% of all osteoporosis medicine prescriptions over the study period.

### Osteoporosis medicine prescriptions for patients

Three-quarters (76.5%) of patients with a recorded diagnosis of osteoporosis also had a record of prescription for osteoporosis medicines (Table 2). These data suggest that up to one quarter of patients with a diagnosis of osteoporosis documented in their files were not prescribed osteoporosis medicines at their regular general practice over a 5-year period.

**Table 2:** Prescriptions for osteoporosis medicines for patients with recorded diagnoses of osteoporosis 2012–17

	Number of patients (*n* = 25,188)	%
**Any osteoporosis medicine**	**19,268**	**76.5%**
*Bisphosphonate*^a^	11,684	46.4%
*Denosumab*	11,762	46.7%
*Other*^b^	2673	10.6%
**No record of osteoporosis medicine**	**5920**	**23.5%**

^a^Bisphosphonate includes alendronic acid, clodronic acid, ibandronic acid, pamidronic acid, risedronic acid, zoledronic acid, etidronic acid, tiludronic acid (either singly or in combination)

^b^Other includes strontium ranelate, tibolone, raloxifene, and teriparatide

### Osteoporosis treatment patterns

Given that denosumab and bisphosphonates are the most commonly prescribed first-line medicines for osteoporosis in Australia, the analysis of treatment initiation and cessation was restricted to patients who were prescribed either denosumab and/or bisphosphonates. Other osteoporosis medicines were not included in this analysis.

#### Initiation of osteoporosis medicines

We analysed the initiation of therapy using denosumab or a bisphosphonate for patients who had a recorded diagnosis of osteoporosis and no record of a bisphosphonate or denosumab prescription in the pre-washout period (*n* = 18,629) (Fig. 1). During the study period 2013–17, a total of 12,091 (64.9%) patients were prescribed therapy with denosumab and/or a bisphosphonate. There were 34.4% of patients who were started on denosumab but not a bisphosphonate, 18.8% who were started on a bisphosphonate but not denosumab, and 11.7% were prescribed both – a bisphosphonate and denosumab – at different times during the study period. Of the patients who were prescribed both a bisphosphonate and denosumab, the majority were prescribed a bisphosphonate before denosumab (82.5%) (Table 3).

**Table 3:** Initiation of bisphosphonates or denosumab in patients with recorded diagnoses of osteoporosis and no record of bisphosphonate or denosumab in pre-washout period 2013–17

	Number of patients (*n* = 18,629)	%
**Prescribed bisphosphonate but not denosumab**	**3502**	**18.8%**
**Prescribed denosumab but not bisphosphonate**	**6407**	**34.4%**
**Prescribed both bisphosphonate and denosumab**	**2182**	**11.7%**
*Prescribed bisphosphonate first*	1800	9.7%
*Prescribed denosumab first*	134	0.7%
*Prescribed bisphosphonate and denosumab on the same day*	248	1.3%

#### Cessation and substitution of osteoporosis medicines

We also analysed patterns of treatment cessation and/or substitution in patients who had a recorded diagnosis of osteoporosis, had at least one recorded prescription of either denosumab or a bisphosphonate during the first 5 years of the osteoporosis treatment period and had ceased treatment, based on the date of last prescription, prior to the post-washout period (Fig. 1). Of the 1122 patients with a recorded diagnosis of osteoporosis, who had been treated with and subsequently ceased denosumab, 218 (19.4%) patients had a record of a prescription for a bisphosphonate following denosumab cessation, therefore 904 (80.6%) patients had no record of a prescription of bisphosphonate either on or after the date of the last denosumab prescription. Of the 7357 patients who had ceased bisphosphonate treatment, 4155 (56.5%) patients had a record of a prescription for denosumab either on or after the date of the last bisphosphonate prescription.

### Phase 2

Interviews were conducted with 13 GPs from MedicineInsight practices in six states in Australia. The eight male and five female GPs had a median of 25 years in practice. Themes mapped to TPB are shown in Table 4.

**Table 4:** Themes arising from the data based on the TPB

GP behaviour based on TPB	Themes arising from the data
GPs’ subjective norms	• Gaps in knowledge
GPs’ attitudes	• Reluctance to start treatment • Perceptions about benefits of treatment
GPs’ perceived behavioural control	• Perceived patient factors – lack of knowledge, low patient adherence • Organisational factors • GP approaches to managing patient factors

### GPs subjective norms: gaps in knowledge

#### Uncertainty about effects of stopping treatment

Most GPs were aware of the quick loss of BMD gains after stopping denosumab and therefore the need for denosumab to be administered every 6 months. GPs, however, expressed uncertainty about when to stop denosumab, what to do when stopping, the risk of stopping denosumab without an alternative being prescribed, or what should be prescribed if a patient had previously had problems with bisphosphonates. As a result of these concerns, GPs often reported referring their patients to specialists for advice about stopping, while others chose not to start denosumab if they thought that the patient was unlikely to return for a follow-up injection.

#### Uncertainty about drug holidays

In general, GPs were aware of the concept of drug holidays, when patients on longer term medication such as bisphosphonates have planned breaks from treatment. Such a holiday was seen as particularly appropriate for patients who had had a good response to bisphosphonates, as indicated by their BMD, and who had not had a fracture after at least 5 years of medication.

Some GPs expressed uncertainty about whether drug holidays were an appropriate approach for their patients, how to undertake drug holidays and what follow-up was required.

“Well, only because I don’t know how long the studies have actually been, you know, how long people have been on these kind of medications. And I understood that you could have a holiday from the bisphosphonates and their effect is much more long-lasting. But like I said, I’ve never felt fully that any – every time I hear a lecture about osteoporosis or whatever, they never ever really say exactly how long people should be on things. Like, it’s pretty vague.” (GP2).

### GPs’ attitudes: reluctance to initiate treatment

#### Perceived lack of urgency of treating osteoporosis

A few GPs expressed the view that, for many patients, there was no urgency in treating this condition with ‘preventive’ medicines and instead chose to periodically review and assess their fracture risk.

“Well, none of these preventative things – they don’t help you, they only really help you if you want to take it for years. There’s absolutely no rush whatsoever in convincing anything like this. Like blood pressure medication, nothing is going to happen next week if you don’t take it.” (GP5).

#### Medicine-related factors and comorbidities

GPs reported hesitancy in commencing medicines in the presence of contraindications or comorbidities such as gastrointestinal disorders, chronic kidney disease, poor renal function, low calcium and vitamin D levels, and poor dentition. They were also concerned about potential side effects of medication.

### GPs’ attitudes: perceptions about benefits of treatment

The GPs were not all convinced about the extent of the effectiveness of osteoporosis medicines and/or their benefit in certain patient cohorts. Some were particularly unclear regarding the time of onset and extent of fracture prevention once a medicine was commenced. While all patients had an ongoing risk of fracture, for some the benefit of altering this risk was perceived to be minimal. This uncertainty often influenced the timeliness of GP prescribing or was the reason for not starting a medicine in the first place. GPs highlighted the need for more information to identify patients at highest risk of fracture who would, therefore, benefit most from treatment.

“I’m a bit reluctant to start the medication unless it’s really indicated because of the potential side-effects … mostly the (rare) bone fractures and other indigestion-type sort of problems. I don’t know if it’s really clear in my mind that people who are … borderline if they benefit from it. It’s only for people I think that are clearly osteoporotic or having fractures.” (GP11).

### GPs’ perceived behavioural control: perceived patient factors

#### Poor patient awareness of osteoporosis and its consequences

GPs considered that most of their patients had low awareness of osteoporosis and its potential impact, which they believed to be a strong contributor to patient non-adherence. This low awareness was particularly attributed to the mainly asymptomatic nature of osteoporosis and the absence of its impact on day-to-day life in its early stages.

“I have a lot of patients would say look, I don’t feel anything, I’ve got no pain, I’ve got no symptoms, why do I need any medication?” (GP8).

#### Patient reluctance to start/continue medicines

GPs perceived that many patients appeared reluctant to start medicines for reasons including scepticism about the need for or benefit of the medicine, concerns about adverse events, medicine administration restrictions and complexity when taking oral bisphosphonates, cost of the medicine and polypharmacy.

“If I could convince them to take it I would but there are some people who just don’t want to take something. And, because they haven’t actually – particularly the ones that don’t have or haven’t had obvious fractures, they don’t feel any ill health, they perceive themselves as active and so on and they don’t want to take tablets. I mean you can’t force people to treat something.” (GP2).

GPs also reported that some patients who had commenced medicines questioned the need to continue them, particularly if they were considered to be harmful or had side-effects. The lack of any tangible benefits of osteoporosis treatment, particularly in the short term, was also cited as a barrier to patient adherence. GPs also mentioned that some patients expressed the desire to take a break from medicines as they did not believe they were at high risk of fracture, were sceptical about the effectiveness of treatment, or had read/heard negative views about the medicines.

“Again, they just have views that it’s harming them and it’s because they realise it’s not [for them] … it may be because they think well, what’s the point, I’ve been taking it and all this time I haven’t had a fracture, I should be right now.” (GP9).

### GPs’ perceived behavioural control: Organisational factors

#### Importance of reminder and recall systems to avoid delaying denosumab

The importance of systems for avoiding delay in administering denosumab was highlighted. Nearly all GPs who regularly prescribed denosumab or zoledronic acid relied on their reminder and recall systems for ensuring their patients were followed up for their subsequent doses at the correct time. However, the absence or deficiencies of such reminder processes described by some GPs meant that some of them relied on patients to request a prescription for denosumab prior to booking a follow-up appointment, potentially leading to a delay in receiving the medicine.

### GPs’ perceived behavioural control: GP approaches to managing patient factors

#### Provision of information on osteoporosis and consequences

GPs acknowledged the need to address patients’ poor awareness of osteoporosis and its consequences though the provision of information on osteoporosis, its diagnosis, consequences such as an increased fracture risk, and why the patient should consider starting an osteoporosis medicine. GPs agreed that sharing good information on osteoporosis is a starting point in the conversation and helps improve patients’ understanding of osteoporosis and of the need for medicines. GPs considered prior fractures, including MTFs, often motivated patients to persist with their treatment to avoid another fracture.

“I think if they understand what they’re trying to prevent and how it can impact on their lifestyle, the loss of independence is a major motivator to keep the treatment up.” (GP12).

#### Patient preference

GPs emphasised the importance of taking patient preferences into consideration when prescribing and noted the need to outline the range of osteoporosis medicines available to patients so they may choose the regimens that match their lifestyles. Patient involvement in the ongoing review process was also deemed important.

“I let them know about the options they’ve got in terms of tablets and once a day, once a week, once a month. Then there’s their injections that are every 6 months and their infusions that are every year. I’d speak to them and maybe gauge their views on some of those things.” (GP3).

## Discussion

This study aimed to identify current osteoporosis treatment patterns and gaps in practice in Australia, using general practice data from the MedicineInsight program and GP interviews. The main findings from the data suggest that, in this population of patients, osteoporosis is underdiagnosed and undertreated. Of concern is the possible discontinuation of denosumab without subsequent antiresorptive therapy.

In our study, the estimated prevalence of a recorded diagnosis of osteoporosis in general practice in patients over the age of 50 was 12%. This proportion increased with age and was higher in women (18%) compared to men (5%), similar to patterns observed in self-reported national survey data [3]. However, the prevalence of osteoporosis diagnosis was lower than estimates of osteoporosis prevalence from Australian population-based studies, which provide objective data on the true prevalence of osteoporosis [3, 25]. This could be explained by a number of factors including under recording of osteoporosis diagnoses or recording of diagnoses in fields not available to MedicineInsight, the lack of access to BMD results and underdiagnosis.

Underdiagnosis of osteoporosis has been reported in the literature, particularly given the often ‘silent’ nature of this condition and the likelihood of diagnosing osteoporosis only when the patient has an MTF [26]. It may also reflect the under-recognition of osteoporosis in general practice and/or a lack of salience of the condition [26]. Indeed, GPs interviewed in our study expressed a lack of urgency in treating osteoporosis with some not recognising the benefit of treating the condition. A previous study investigating health provider views around post-menopausal osteoporosis reported similar findings, with providers often trivialising post-menopausal osteoporosis, disqualifying it as a legitimate disease and displaying a lack of urgency in diagnosing or treating it [27]. Further, MTFs and complications were described as infrequent, limited in time and ‘repairable’, and the increased mortality risk was downgraded as post-menopausal osteoporosis was perceived to be unlikely [27].

The general practice data in our study showed a steady increase in the proportion of osteoporosis medicines prescriptions over the study period 2012–17, with a specific increase in the number and proportion of denosumab prescriptions. Despite the increase in the number of osteoporosis prescriptions over the study period, almost a quarter of the patients with a recorded diagnosis of osteoporosis did not have any record of a prescription for an osteoporosis medicine – suggesting potential undertreatment of the condition within this cohort. It is possible that osteoporosis treatment may not be indicated in these patients based on their individual risk factors or may be declined by these patients [1]. Although we did not investigate risk factors for osteoporosis or reasons for non-prescription, several other Australian studies have demonstrated suboptimal rates of treatment of osteoporosis in primary care, even for patients with significant risk factors [26, 28–31]. An audit of 10 GPs in an Australian rural practice, extracting data on patients with a risk factor for osteoporosis (> 60 years, *n* = 420) over a 12-year period, reported that 26% of patients diagnosed with osteoporosis were not receiving any treatment [28]. A more recent study using electronic medical records data to identify rates of osteoporosis in patients aged over 70 years reported the absence of a recorded current treatment prescription for 29% of patients with osteoporosis [26]. Further, even the presence of major osteoporotic risk factors, including prior fractures, did not affect the likelihood of investigation or treatment of osteoporosis in general practice [26, 31].

Analysis of the qualitative data using the TPB revealed GP beliefs and attitudes that may have influenced GPs’ intentions towards osteoporosis management, leading to the suboptimal treatment rates shown in our study. The study explored GP beliefs and suggests a lack of clinical knowledge about ceasing osteoporosis treatment, especially for denosumab, and about the appropriateness of drug holidays, especially for bisphosphonates. GPs’ attitudes towards the benefits versus risks of osteoporosis treatment and the non-urgency of treating the condition were identified. We explored GPs’ perceived control beliefs, including patient factors such as low awareness of osteoporosis and its impact, as well as, the cost and side effects of treatment. Some of these factors, especially the knowledge gaps around therapeutic management of osteoporosis, have been identified in previous studies [27, 31]. We hope that the identification of these factors influencing GP intentions to prescribe will help inform the development and implementation of specific interventions to improve the treatment of osteoporosis, such as clinical education programs addressing GP knowledge gaps and attitudes in osteoporosis treatment, especially in the light of relatively newer drugs such as denosumab.

This study demonstrates an increase in the proportion of denosumab prescriptions during the study period. The recent evidence demonstrates improved patient preference for and adherence to 6-monthly subcutaneous denosumab injections compared to oral bisphosphonates [32, 33]. In addition, Australian guidelines recommend denosumab as a first line treatment as well as other treatments [6].

Our study suggests that there is insufficient substitution with another osteoporosis medicine when ceasing denosumab in general practice patients. More than 80% of the patients who had stopped denosumab received no subsequent prescription for a bisphosphonate, potentially exposing them to rapid bone loss. Cessation of denosumab (and hence the loss of the drug’s effect on bone remodelling) leads to rapid bone loss. There have also been case reports of multiple vertebral fractures occurring shortly after stopping denosumab [17, 34]. A recent post hoc analysis of the denosumab phase 3 randomised trial (FREEDOM) and its extension study demonstrated a small but significant increase in the risk of multiple vertebral fractures after cessation of denosumab, particularly in patients with prevalent vertebral fractures [35]. All this highlights the need to consider switching to another osteoporosis therapy if denosumab is discontinued, to mitigate the potential for rapid bone loss [6, 36].

Similar to denosumab, bisphosphonates were also stopped without any recorded follow-up treatment being substituted for some patients. We cannot tell from the data the reasons for the failure to continue either medicine, but it is likely that patient non-adherence contributes to these figures, in that some patients will choose to stop their medicines without discussion or follow-up. The factors leading to non-adherence of osteoporosis medicines are complex [15]. Some of the main patient factors affecting osteoporosis management perceived by interviewed GPs were poor patient awareness of the potential impact of osteoporosis, concerns about side effects prior to starting treatment and actual side effects once started, lack of obvious benefits while on treatment, negative impressions of medicines from the media or friends/family, and cost. It is important to check for adherence at every consultation. Adherence may be improved through patient education, and pharmacist support as well as shared decision making when appropriate [37]. Other potential reasons for ceasing either medicine could be GP preference to cease treatment or lack of efficient recall systems particularly for patients having 6-monthly denosumab injections.

The interviews indicated that GPs are uncertain about the effects of stopping denosumab and ways to approach treatment, particularly if a bisphosphonate had previously been trialled. This potential uncertainty and lack of knowledge around denosumab could be attributed to the fact that denosumab is a relatively new medicine for many GPs, compared to bisphosphonates. Education about osteoporosis treatment alone is unlikely to be sufficient as we found other factors that appeared to impact on GP intentions to prescribe. Such factors influencing adoption and prescribing of new drugs have been previously highlighted in the literature including patient factors, drug characteristics, recommendation from peers and experts, clinical guidelines, pharmaceutical marketing and familiarity with the therapeutic area and knowledge of the drug [38]. We feel that targeted interventions may help to improve prescribing and adherence. It is important that general practices use good reminder/recall systems and consider the sharing of electronic records across the health system, namely ‘My Health Record’ which has recently been introduced in Australia, to improve the overall care and management of osteoporosis in the community [39].

### Strengths and limitations

Patients within the MedicineInsight cohort are broadly representative of the Australian population in terms of patient demographics and rates of disease. We have adopted a mixed methods approach, and our qualitative findings support the trends in osteoporosis treatment observed using quantitative MedicineInsight data. The latter cover a substantial segment of the population seen in general practice and potentially indicate undertreatment of osteoporosis. In this context, it is important to note that the present study analyses treatment initiation only in patients who already had a recorded diagnosis of osteoporosis. As many patients with osteoporosis go undiagnosed, this study looks at a subsegment of the total population of men and women with recorded osteoporosis. Based on current guidelines, three-quarters of patients with a GP-recorded diagnosis of the disease are being treated with osteoporosis medicines [6]. Our quantitative analyses indicate that the majority of patients who cease denosumab do not receive maintenance therapy, potentially leaving these patients at risk of bone loss.

This study has limitations. MedicineInsight data depend on the accuracy and completeness of the data recorded in the CISs and can vary in quality. Data in CISs are entered with the primary purpose of managing and providing care for patients and not specifically for the purpose of research. This may have led to under-reporting of osteoporosis identification, depending on GP recording practices. Our definition of osteoporosis diagnosis was based on commonly accepted definitions, but there are likely to be variations in how GPs record a diagnosis of osteoporosis. Due to confidentiality issues, the data extraction could not extend to GP progress notes, which may have contained further information on diagnoses and reasons for prescriptions or encounters. Prescription counts may have been an overestimate of actual dispensed prescription counts, as presumably not all prescriptions and repeats will have been dispensed although our analyses excluded repeat prescriptions. This study was unable to retrieve data of patients who receive prescriptions from GPs at non-MedicineInsight practices, or from specialists and other services, and was our best approximation of treatment initiation for our cohort. It must also be noted that, in Australia, patients do not register with a single general practice and are free to visit multiple practices of their choice. This study assumed that patients who had no record of a prescription for an osteoporosis medicine during the 1-year pre-washout period had not taken osteoporosis medicine prior to the study. The qualitative study had a small sample size, which may have affected the trustworthiness of the findings. The qualitative study was designed to elicit GPs’ perspectives and did not seek to address patient perspectives directly.

## Conclusion

This study suggests that within the Australian general practice setting, osteoporosis is underdiagnosed and undertreated. Most patients who had ceased denosumab treatment had no record of a subsequent prescription for another anti-resorptive agent to prevent rapid bone loss usually seen after ceasing denosumab therapy. GP interviews indicated GP beliefs and attitudes that may have influenced their intentions towards osteoporosis prescribing and management. The study suggests the need for clinical education programs addressing GP knowledge gaps and attitudes in osteoporosis treatment, especially in the light of relatively newer drugs such as denosumab. In addition, the study highlights the need for implementation of specific interventions such as reminder/recall systems and further exploration of the effects of GP and patient shared decision making processes.

## Supplementary information

**Additional file 1.** Interview guide: Interview questions for GPs.

## Data Availability

The current study is based on data from MedicineInsight (https://www.nps.org.au/medicine-insight), a national general practice data source developed and managed by NPS MedicineWise. In accordance with the ethical approvals for this study, on-provision of these data is not permitted as it would compromise the patients’ confidentiality and participants’ privacy. However, other researchers would be able to access these data in the same manner as the authors. Data access enquiries can be directed to NPS MedicineWise (medicineinsight@nps.org.au).

## Abbreviations

BMD: Bone Mineral Density
CI: Confidence Interval
CIS: Clinical Information System
GP: General Practitioner
MTF: Minimal Trauma Fracture
PBS: Pharmaceutical Benefits Scheme
RACGP: Royal Australian College of General Practitioners
SEIFA: Socio-Economic Indexes for Areas
TPB: Theory of Planned Behaviour
