# Supplementary material for: Osteoporosis management in Australian general practice: an analysis of current osteoporosis treatment patterns and gaps in practice
Source: BMC Fam Pract. 2020 Feb 12;21:32. doi: 10.1186/s12875-020-01103-2 (PMC7014771; doi:10.1186/s12875-020-01103-2)
Supplement: Supplementary file 1 — Additional file 1. Interview guide: Interview questions for GPs. [file 12875_2020_1103_MOESM1_ESM.docx]

**Interview questions for GPs**

| ***Choice of osteoporosis medicine***   - On average, how many osteoporosis-related encounters do you provide per month? - Do you usually commence osteoporosis medicines for your patients? - If no, who usually starts osteoporosis medicines for patients managed by you? (e.g. other GPs, specialists, started in hospital) |
| --- |
| ***GP approach to starting medicines***   - How would you usually discuss starting an osteoporosis medicine with patients? - Which medicines do you commonly prescribe? And why? - *Medicine available on PBS include;* - *Alendronate once weekly* - *Risedronate once daily* - *Risedronate once a week* - *Risedronate once a month* - *Zoledronic acid* - *Denosumab* - *Raloxifene* - *Teriparatide* - *Other________________* - What influences your choice of therapy when starting treatment? - Does patient preference play a role in your decision to choose a specific medicine? - Does your choice of treatment differ across patients? - Are there any reasons why you may not start a medicine for a patient? - Do your patients voice any particular concerns about their condition or about commencing treatment? - What questions do patients tend to ask you about their medicines? - What side/adverse effects are patients particularly concerned about? - How would you address patient concerns around the possible benefits vs risks of medicines? |
| ***GP approach to stopping/ceasing medicines***   - Are there any situations where you might decide to switch a medicine for a patient who has been taking it for few years? - Are there any specific situations where you advise patients to stop their osteoporosis medicines completely? - Do you consider or recommend pausing treatment (“drug holidays”) for patients who have been on treatment for years (e.g. on bisphosphonates). And why? - Are there any osteoporosis medicines for which you would not consider a drug holiday? If Yes, which are those and why? - What management strategies do you suggest/prescribe for patients recommended to stop their medicine? (Other medicines, complementary medicines, lifestyle measure) - Do you consider restarting treatment again at a later stage? If yes, based on what data/circumstances and when? Would you restart the same or different medicine? |
| ***GP views on patient adherence***   - In your experience, how likely are patients to adhere to their osteoporosis medicines? What motivates patients to adhere to their osteoporosis medicines? - In your opinion, which patient cohorts are more likely to be non-adherent? Are there specific factors or reasons that contribute to patients being non-adherent to treatment? - How does adherence appear to differ across different medicines? (I.e. is it different for oral vs injectable?) - In your experience, do you commonly observe patients stopping their treatment completely? If yes, what do you think are some of the reasons why patients stop treatment completely? - Do you commonly observe patients not starting their treatment immediately? If yes, what do you think are some of the reasons why patients may delay their treatment? |
| ***GP approach to managing non-adherence***   - Do patients typically advise you when they wish to stop or delay their medicine? If yes, how do you respond? - How do you manage patients who are consistently non-adherent to treatment? - What is your approach to encouraging patients to adhere to their osteoporosis medicines? - Do you use any tools or resources to encourage patients to adhere to their medicines? - Do you implement any specific strategies to encourage patients to restart/continue their medicines? Do these strategies appear to work for your patients? - Do you discuss the risk of fractures with patients who stop their medicines? |
| ***Other questions***   - If NPS MedicineWise was to develop an educational program for GPs that aimed to address adherence to osteoporosis medicines, what would you like to see in this program? - Is there anything else you would like to comment on in relation to osteoporosis medication and adherence? |
